# Compliance with smoke-free laws in hospitality venues in Ethiopia: qualitative insights into barriers and facilitators using the tobacco control theoretical framework

**DOI:** 10.1186/s12889-025-26012-w

**Published:** 2025-12-20

**Authors:** Selamawit Hirpa, Noreen Dadirai Mdege, Terefe Gelibo Argefa, Selam Abraham Kassa, Wakgari Deressa

**Affiliations:** 1 Department of Epidemiology and Biostatistics, School of Public Health, College of Health Sciences, Addis Ababa University, Addis Ababa, Ethiopia; 2 Department of Health Sciences, University of York, York, YO10 5DD UK; 3 Centre for Research in Health and Development, York, UK; 4 Development Gateway: an IREX Venture, Washington, DC United States of America; 5 Aklilu Lemma Institute of Health Research, Addis Ababa University, Addis Ababa, Ethiopia

**Keywords:** Smoke-Free laws, Tobacco control, Compliance, Hospitality venues, Qualitative study, Ethiopia

## Abstract

**Background:**

Ethiopia has made significant strides in adopting comprehensive smoke-free laws aligned with the World Health Organization’s Framework Convention on Tobacco Control, which mandates that all public places, including hospitality venues, public transportation, and workplaces, be 100% smoke-free. However, effective implementation across regions remains uneven. This study examines the barriers and facilitators influencing the enforcement of smoke-free legislation in Ethiopia.

**Methods:**

This qualitative study was conducted in 10 major cities in Ethiopia, involving 41 key informant interviews with individuals working on tobacco control. For this study, a framework comprising five key concepts, namely, institutions, agendas, networks, socioeconomic conditions, and ideas, was employed. Semi-structured interview guides were developed in English and then translated into local languages. All audio-recorded interviews were transcribed verbatim and translated into English. Deductive thematic analysis was conducted using ATLAS. Ti Scientific Software.

**Results:**

The study identified several key facilitators of compliance with smoke-free laws in Ethiopia, including a well-established legal framework, clear institutional structures, especially in the capital city (Addis Ababa), strong political leadership, stakeholder collaboration, supportive cultural and religious norms, and growing public awareness of the harms associated with tobacco use. However, significant barriers remain, particularly in regional areas. These include the absence of dedicated tobacco control units, lack of region-specific implementation guidelines, competing policy priorities, limited budget and human resources, and insufficient law enforcement engagement. Cultural acceptance of smoking in some communities, economic reliance on tobacco farming, and misconceptions about products like shisha further undermine compliance with smoke-free laws. Additionally, weak coordination across sectors and limited ownership of tobacco control responsibilities among non-health actors hinder effective implementation.

**Conclusion:**

The study highlights the need for region-specific guidelines, accountable institutions, improved coordination, targeted education, and the addressing of socioeconomic barriers. Leveraging existing strengths and addressing identified challenges will be critical to achieving equitable and effective enforcement of smoke-free laws nationwide.

**Supplementary Information:**

The online version contains supplementary material available at 10.1186/s12889-025-26012-w.

## Introduction

There is no safe level of second-hand tobacco smoke (SHS) exposure, and even brief exposure can cause harm [1]. Ethiopia has a relatively low smoking prevalence, but the absolute number of smokers remains high due to the country’s large population size [2]. According to the Ethiopia Global Adult Tobacco Surveys (GATS) conducted in 2016 and 2024, more than 20% of non-smokers who visited restaurants were exposed to second-hand smoke (SHS) [3, 4]. Smoke-free laws play a critical role in protecting the public from the harmful effects of SHS and in promoting a smoke-free culture [5, 6].

The World Health Organisation Framework Convention on Tobacco Control (WHO FCTC) mandates that all public places, including hospitality venues, public transport, and workplaces, be 100% smoke-free [7]. In alignment with this, Ethiopia has enacted one of the strongest comprehensive tobacco control proclamations in Africa, mandating 100% smoke-free public places and workplaces [8]. However, the presence of strong laws does not automatically ensure compliance.

Observational studies in multiple cities in Ethiopia reveal that active smoking is still prevalent in indoor spaces, especially in bars, nightclubs, and certain regions like Afar and Gambella, where compliance was notably poor [9]. Only a small fraction of venues fully adhere to smoke-free requirements, and the presence of ‘no smoking’ signage is limited, further undermining the effectiveness of the law [9, 10]. Studies measuring indoor air quality in hospitality venues in Addis Ababa found that most establishments have particulate matter (PM_2.5_) concentrations far exceeding the WHO [11], indicating significant exposure to SHS [12]. SHS exposure is particularly high in venues where shisha and cigarette smoking are observed, underscoring the urgent need for stricter enforcement and public education on the risks of SHS. Several factors have been identified as contributing to poor compliance with smoke-free laws in Ethiopia. These include a lack of knowledge about existing laws, unfavourable attitudes toward legislation, weak enforcement mechanisms, limited institutional capacity, political instability, and poor coordination among stakeholders [10, 13, 14]. Studies conducted in Africa and Asia have also identified several barriers to the implementation of smoke-free laws, including fear of financial impact, interference from the tobacco industry, inadequate resources, negative customer attitudes, unclear institutional roles, and weak enforcement mechanisms [15–17].

Previous studies conducted in Ethiopia have predominantly used quantitative methods, identifying both successes and gaps in compliance with smoke-free laws. However, there remains a critical gap in understanding why compliance with smoke-free laws was successful in some settings and unsuccessful in others. Qualitative research, particularly through key informant interviews (KIIs), is needed to uncover the underlying reasons for these variations and to explore the context-specific factors that influence the effective implementation of smoke-free policies. To date, no qualitative study has examined the barriers and facilitators to implementing smoke-free laws in Ethiopia. This study aims to explore. barriers and facilitators influencing compliance with smoke-free laws in Ethiopia.

## Methods

### Study setting

This study was conducted across 10 purposively selected major regional and chartered cities in Ethiopia, including Addis Ababa, Adama, Assosa, Bahir Dar, Dire Dawa, Gambella, Harar, Hawassa, Jigjiga, and Semera, representing diverse geographic regions, administrative structures, and population demographics. These cities were purposefully selected to capture a range of urban contexts and regional variations in the implementation and enforcement of smoke-free laws, as well as cultural practices related to tobacco use.

### Theoretical framework for the study

We adapted the five main explanatory factors (institutions, agendas, networks, socioeconomic factors, and ideas) proposed for tobacco control [18, 19] and was recently used to evaluate the implementation of the tobacco control policy in Indonesia [19]. Institutions are administrative organisations responsible for formulating and implementing tobacco control-related policies. In Ethiopia, this includes multi-level governmental institutions, ranging from the central government, which is heavily involved in policy formulation, to implementers at the grassroots level of the administrative structure. Institutions also include non-governmental actors, such as international organisations, involved in tobacco control. Agendas refer to how tobacco is framed and whether it has received the necessary attention as a policy issue.

Networks identify coordination between groups, organisations, and people who have a vested interest in smoke-free laws policy and enforcement. Socioeconomic factors encompass the cultural and social norms within communities that shape attitudes toward tobacco use and influence compliance with smoke-free laws. Economic factors also include financial constraints or interests, such as dependence on tobacco for livelihoods or business-related concerns, that limit the public’s willingness to enforce laws. Ideas include the role of knowledge and beliefs in smoke-free compliance, as well as the public’s and stakeholders’ knowledge and beliefs about smoke-free laws.

### Sampling approach

A total of 41 key informants (KIs) were identified from organizations involved in tobacco control in Ethiopia. The initial plan was to interview 2 to 3 participants from each of the nine regional cities and approximately 10 participants at the national level. The final number of KIIs was guided by insights from the daily debriefing sessions and reached when no new information emerged, indicating data saturation. Interviewees were selected at both national and regional levels in consultation with the Ethiopian Food and Drug Administration (EFDA), the lead organization for tobacco control and chair of the national tobacco control committee. Lists of regional tobacco control focal persons were obtained, who further assisted in identifying additional key informants working on tobacco control in their respective regions. For the nine regional cities, individuals were from the health bureau, the regulatory body, and the Ministry of Trade at the regional/zonal/district level. In Addis Ababa, KIIs were conducted with individuals from the Ministry of Health (MoH), Ethiopian Food and Drug Authority ( EFDA) Ministry of Trade and Industry, WHO, National Tobacco Control Coordination (NTCC), the Campaign for Tobacco-Free Kids (CTFK), Addis Ababa University (AAU), Ethiopian Public Health Institute (EPHI), Ethiopian Thoracic Society, Addis Ababa Health Bureau, Addis Ababa Food, Medicine and Health Care Administration and Control Authority (FMHACA). and civil society organisations (CSOs) such as Mekuamia, and the Mathiwos Wondu Ethiopian Cancer Society.

### Data collection

Semi-structured interview guides were developed in English (see Supplementary File 1), translated into Amharic, Afan Oromo, and Somali, and then back-translated into English to ensure consistency and accuracy. The guides were designed to explore participants’ perspectives on the facilitators and barriers to compliance with smoke-free laws. One research assistant was deployed in each study city, except in Addis Ababa, where two were assigned due to the presence of key informants at both national and regional levels, including representatives from international organisations. Interviews were conducted in local languages, lasted 40–90 min, and were audio-recorded. Transcriptions were produced in the original language and subsequently translated into English. Field notes summarizing key points were prepared after each interview. Data were collected between December 9, 2022, and January 5, 2023.

### Data verification and validation procedures

Credibility was strengthened by recruiting 11 experienced qualitative research assistants, who were academic staff from universities, and by providing them with 2 days of training on the study guides and data collection procedures. After each interview, research assistants confirmed key points with participants. All interviews were audio-recorded and transcribed verbatim by trained assistants to ensure accuracy. A sample of audio recordings was compared with their transcripts to ensure transcription accuracy. Independent transcribers and coders reviewed transcripts multiple times during codebook development. Sample transcripts were jointly coded, and iterative discussions with the research team were used to refine and validate the codebook. Two coders independently coded each transcript, and regular discussions were held between the coders and the broader research team to resolve discrepancies and ensure analytical consistency. The research team maintained reflexivity throughout the study to ensure that prior experiences did not unduly influence interpretation and that interpretations were grounded in the data. The research team carefully reviewed the entire analytical process to maintain methodological rigor, chronological accuracy, and a balanced understanding of the findings.

### Data analysis

We used a deductive coding approach guided by the study’s theoretical frameworks. All transcribed and translated transcripts were read in full and uploaded to ATLAS.ti Scientific Software Development GmbH [ATLAS.ti v3.15.0] (https://atlasti.com*)* (2022). Text segments were coded using a combination of deductive codes derived from the framework and inductive codes emerging from the data. While primarily deductive, the process remained flexible, allowing the addition of new subcodes when data did not fit the existing framework, thereby enabling systematic organization and alignment of findings with the predefined thematic areas.

## Results

The results are based on data from 41 key informants in the capital, Addis Ababa, and nine major regional cities. The facilitators and barriers to compliance, monitoring, and enforcement of smoke-free laws are presented below under the five main explanatory factors, i.e., institutions, agendas, networks, socioeconomic factors, and ideas.

### Facilitators of compliance with smoke-free laws

As shown in Table 1, facilitators of compliance with smoke-free laws often include a combination of organisational structure, the presence of tobacco control laws, prioritisation of smoke-free laws on the agenda due to strong leadership and commitment, stakeholder engagement through networks, supportive cultural norms and religious views, and public awareness of the laws.

**Table 1:** Barriers and facilitators to compliance with Smoke-Free laws

Theoretical framework for tobacco control	Facilitator definition (Illustrative examples)	Barrier definition (Illustrative example)
Institution	**Organizational structure** -Well-established organisational structure in Addis Ababa to monitor compliance with smoke-free laws. *-* Toll-free numbers to report the breach of smoke-free laws.	**Organizational structure** -Lack of specific units responsible for managing tobacco control programs in most study areas -All tobacco control focal persons have additional non-tobacco-related assignments
	**Tobacco control laws and directives** -Ethiopia has a comprehensive and strong tobacco control law, which is Proclamation [1112/2019]	**Tobacco control laws and directives** *-*Regions lack specific tobacco control laws and directives.
Agenda	-At the national level, there was a strong political leadership and commitment for compliance and monitoring of smoke-free laws. *-*Smoke-free law was a priority agenda for the government, civil societies, and NGOS	-Smoke-free laws were not prioritized as a key issue in some regions, and non-health departments *-* Some regions had other priority agendas, like crime and security issues.
Networks	-There was a strong stakeholders’ engagement to comply with and monitor smoke-free laws, especially among those working in health areas *-*The lead organisation (FDA) networks with NGOs, government bodies, and civil societies	-Lack of coordination at departments beyond health, for example, law enforcement agencies outside the health sector often do not view tobacco control as part of their mandate.
Socioeconomic factors	**Culture**,** societal norms**,** and religion** *-*Smoking is not considered a positive behaviour in most cultures and religions.	**Culture**,** societal norms**,** and economic factors** -Use of traditional tobacco products or smoking was considered part of the culture in some regions. -Hospitality venue owners are afraid to lose customers if they comply with smoke-free laws. -Bribery of law enforcers by hospitality venue owners to avoid smoke-free law compliance
Ideas	-The public’s knowledge about the smoke-free laws had improved, especially in the Capital city, Addis Ababa -Capacity building programs regarding smoke-free laws were organized for the hospitality venue owners and managers at the capital city	-Lack of knowledge and negative perception about the smoke-free laws. Moreover, not consider shisha as a tobacco product.

### Institution

#### Well-established organisational structure

Key informant participants discussed the existence of a clear structure in place to monitor the implementation of smoke-free laws. This was viewed as especially true for Addis Ababa (the capital city), where the regulatory structure extends to the district level, giving the regulatory body full capacity to facilitate effective implementation, monitoring, and enforcement. Hospitality venues also rely on the regulatory body to renew their licences, thereby increasing the likelihood that they will comply with the regulations. Furthermore, the availability of toll-free numbers in some cities to report breaches was considered to have contributed to the successful monitoring and enforcement of the law.

An interviewee mentioned that: *Hospitality venue licenses are renewed under the EFDA structure at the district level*,* meaning that non-compliance with smoke-free laws or other standards results in the license not being renewed. This proximity offers an opportunity for enhanced monitoring and law enforcement activity.*

[Tobacco control expert at the national level]

### Tobacco control laws and directives

Most study participants agreed that Ethiopia has strong tobacco control laws, specifically Proclamation [1112/2019] and Directives [771/2021]. This legal framework provides a solid foundation for implementing tobacco control laws in Ethiopia. Moreover, it was repeatedly mentioned that the proclamation clearly defines the roles of law enforcement officers in enforcing smoke-free laws.

A participant stated that:

*Previously*,* the laws did not prohibit smoking in public places*,* making it impossible to ask someone to refrain from smoking. However*,* with the new proclamation*,* we are now empowered to stop individuals from smoking in public*,* as the law provides the necessary support.*

[Tobacco control expert from the Region]

Another interviewee stated that:

*T* *he smoke-free laws stipulate that those who violate the law will face penalties in Ethiopia, and law enforcers have a clearly defined role in ensuring compliance.*

[A study participant from the region]

### Agenda

#### Political leadership and commitment

Study participants described the implementation of smoke-free laws as a top priority for the federal government and for some regional governments, as well as for NGOs and civil society organizations involved in tobacco control. This commitment was demonstrated through the creation of platforms for continuous monitoring of smoke-free laws, efforts to engage the tobacco industry to remove implementation barriers, and the allocation of necessary resources. Strong political leadership and commitment were highlighted as pivotal factors in the effective monitoring and enforcement of laws, particularly in Addis Ababa, where high-level officials have established multiple platforms to support these efforts.

An interviewee stated that:

*To monitor the implementation of the smoke-free law*,* the Addis Ababa FMHACA higher official created a Telegram group*,* and reports would be uploaded daily from the district to the sub-city. The sub-city would share the reports with the Addis Ababa FMHACA central office weekly. The management committee would evaluate the implementation of the smoke-free law weekly.*

[An interviewee from civil society, national level]

Another interviewee explained that:

Being a priority area also enabled the allocation of resources to the issue, as indicated by a statement from an interviewee below:

*…. because it [smoke-free laws implementation] is a priority area*,* the government and civil society organisations have spent a lot of resources.*

[A tobacco control expert from addis Ababa]

### Networks

#### Stakeholder engagement

Participants discussed that the lead institutions for the tobacco control network collaborate with other local and international organisations to implement, monitor, and enforce smoke-free laws.

An interviewee stated that:

*According to the 2016 survey*,* tobacco use was highest in our region. As a result*,* we received financial support from stakeholders*,* totalling $34*,*000 for a one-year smoke-free project we implemented. We created a 12.4% smoke-free environment.*

[An interviewee from the Regional Health Bureau]

Another participant mentioned that:

*There are more than twenty government offices represented in the current tobacco control committee*,* including the Road Transport*,* Sport Commission*,* Customs*,* Media*,* Culture*,* and Tourism*,* among others*,* which are considered stakeholders*,* and we are working together. These are the organisations that support us by educating the public about smoke-free laws*.

[Regulatory body from the region]

### Socioeconomic factors

#### Culture, societal norms, and religion

Tobacco smoking is not considered a positive behaviour in most cultures and religions, with exceptions in some areas, which is very supportive of compliance with smoke-free laws.

A Study participant mentioned that:

*In some cultures*,* it is considered shameful to have a cigarette……. And in most religions*,* smoking is forbidden. If he is a smoker*,* he will be advised not to do it*,* and if he can’t stop*,* he might be banned from coming to church”.*

[Interviewee from the region]

### Ideas

#### Knowledge and public awareness

Stakeholders invested effort in increasing public awareness about smoke-free laws, which facilitated their effective implementation. Even though there are variations in the level of public and staff awareness between cities and institutions, respectively.

An interviewee mentioned that:

*Capacity-building work has been conducted for hospitality owners and managers*,* especially in Addis Ababa.*

[A study participant from the national level]

### Barriers to smoke-free laws compliance at hospitality venues

Barriers to implementing smoke-free laws include the absence of a dedicated tobacco control department, the lack of implementation guidelines, not being prioritized on the agenda, cultural norms that encourage tobacco use, limited awareness of smoke-free laws, economic constraints, and weak coordination, as shown in Table 1.

### Institution

#### Lack of a department solely focused on tobacco control

Most participants agreed that there is a lack of specific units responsible for managing tobacco control programs at both the federal and regional levels. Tobacco control staff often have other competing responsibilities. Any additional tasks could undermine their work, given the extensive efforts required to implement and monitor smoke-free laws. Furthermore, in most regions, the tobacco control unit is housed under the regional health bureaus, which limits the implementation, monitoring, and enforcement of smoke-free laws.

An interviewee stated:

*To my knowledge*,* regulatory bodies are tasked with monitoring compliance with smoke-free laws in hospitality venues*,* even though they also have other non-tobacco-related responsibilities. The tobacco control program should operate as an independent institution because it requires significant resources and expertise.*

[Tobacco control expert, National level]

Another interviewee stated:

*In our region*,* the tobacco control program falls under the health office as a single directorate. We strongly believe that this directorate should be an independent institution.*

[Tobacco control focal person, regional level]

### Lack of implementation guidelines/directives

Another barrier to effective implementation and enforcement of smoke-free laws was the absence of regional regulations and implementation guidelines. Participants mentioned that regions should adopt strong national tobacco control laws to enable the development of implementation guidelines and the allocation of budgetary resources.

An interviewee stated:

*The regions should adopt their tobacco control laws. The regional implementation law should be passed by the regional parliament*,* which is essential. The national minimum standard has been set*,* but the regions develop their annual plans and shape their organisational structures based on regional laws.”*

[Interviewee from civil society at the national level]

Another interviewee said:

…*our weakness is that the tobacco control law [Proclamation 1112/2019] has not been adopted in our region.*

[Interviewee from the Regional Health Bureau]

### Agenda

#### Not being prioritised on the agenda

Smoke-free law implementation, monitoring, and enforcement are indeed priority agendas for the government and the lead organisations in tobacco control programs. However, this is not always true at the regional level or in departments beyond the health sector. Participants cited several reasons, including crime, security, and market inflation. Activities related to monitoring and enforcement of smoke-free laws are very demanding and broad. Due to overlapping responsibilities, some tobacco control tasks have been neglected. Moreover, the health effects of tobacco use are not immediately visible, which contributes to the lack of prioritisation by regulatory bodies.

An interviewee said:

*Law enforcement officers have a demanding workload. Now we are not only dealing with tobacco*,* but we are also working on other things… Yes*,* there is pressure… In a year*,* we don’t accomplish 30% of what we planned.*

[Interviewee from the Regional Trade and Industry Office]

Another interviewee stated:

… *When we look at the security of the country and our city*,* there is a lot of crime and many problems*,* so now the security agency is paying attention to those issues. But when you talk about smoke-free laws implementation and monitoring*,* or general tobacco control implementation… they say*,* ‘Why are you talking about that?’ There is no reason the security structure can monitor this issue because there are other agendas that are bigger than this [smoke-free laws implementation]. They are not paying attention to this issue*.

[Interviewee from the Regional Trade and Industry Bureau]

Another interviewee mentioned:

*It is challenging to monitor smoke-free law implementation at nightclubs… Second*,* most users typically go to nightclubs at night… Nightclubs are very difficult*,* and it is also unclear to me how they will be monitored. Honestly*,* we never inspected at night.”*

[Tobacco control focal person from the region]

### Socioeconomic factors

#### Culture and societal norms

In some regions, the use of tobacco products is integrated into multiple cultural practices, and law enforcement officials are also influenced by these cultural norms, which pose challenges for the implementation, monitoring, and enforcement of smoke-free laws. Moreover, in some areas, khat chewing, which is associated with smoking behaviour, has become normalised. Moreover, multiple participants reported that law enforcement officers themselves sometimes violate the very laws they are supposed to enforce. This is especially true in a society where tobacco smoking is the norm.

An interviewee stated:

*…culture, which is something you have been immersed in for a long time, and it is difficult to stop [tobacco smoking].*

[Tobacco control expert at the regional level]

Another participant explained:

*Most of the police forces are smokers… Extensive work is still required. The reason is that if you tell the policeman to take the smoker to the security department, but the policeman himself smokes in a public place, it is not practical.*

[Regulatory body, from region]

#### Economic reasons

Participants reported that in the southern part of Ethiopia, many farmers are currently growing tobacco. This has contributed to increased tobacco use, posing challenges for the implementation of smoke-free laws. In addition, hospitality venue owners do not want to lose customers due to smoking restrictions. If they prohibit smoking, customers might go to other venues.

An interviewee said:

*Now*,* many people produce tobacco plants. For example*,* there is a factory in Blate [name of the place] that offers farmers financial incentives to grow tobacco. The farmers also grow tobacco to support their livelihoods. The second issue is that smoking is addictive…”*.

[Tobacco control expert from the region]

Another interviewee said:

*Hospitality venue owners disagree about creating a smoke-free environment… The investor is more concerned about their business than the damage caused by smoking.*

[Interviewee from the Regional Health Bureau]

### Ideas

#### Knowledge and perception

Study participants reported that, although the law is clear, a significant knowledge gap exists among both the general community and law enforcers outside the health sector, posing challenges for implementation. Furthermore, participants frequently raised that shisha is not considered a tobacco product and is becoming more popular in urban settings, creating obstacles for law enforcement. Participants repeatedly mentioned that the public often asks why the government does not completely ban tobacco use and the tobacco industry, rather than just prohibiting smoking in hospitality venues. Some hospitality venue owners and customers also lack a positive attitude toward implementing smoke-free laws.

An interviewee said:

…*the issue of shisha has been neglected… Society does not perceive shisha as a tobacco product*,* and there is a knowledge gap. Even celebrities use it [shisha] for beauty*,* and the same is true for the community… Shisha is becoming a fashion*,* especially in Addis Ababa.”*

[Interviewee from Addis Ababa]

### Networks

#### Lack of coordination

Law enforcement officers outside the health sector have not been actively involved in smoke-free law monitoring and enforcement due to limited awareness and a lack of perceived responsibility for this task.

A study participant discussed:

*The police do not have a system to handle this… They don’t own it; they consider monitoring and enforcement of smoke-free laws the responsibility of others [health departments].*

[Regulatory body from the region]

## Discussion

This study explored both facilitators and barriers to the implementation of smoke-free laws in hospitality venues across Ethiopia, analysing them through a tobacco control framework that focuses on institutional structure, agendas, socioeconomic factors, networks, and ideas and perceptions. This framework is particularly appropriate for Ethiopia because it captures both policy-level and contextual factors that shape tobacco control implementation.

At the institutional level, Ethiopia benefits from a well-established legal and organisational framework for tobacco control, which clearly defines roles and penalties related to smoke-free environments. In cities like Addis Ababa, the regulatory structure extends down to the district level, facilitating effective licensing, monitoring, and enforcement. Studies conducted in other countries also showed that tying hospitality licenses to compliance with smoke-free laws empowers regulatory authorities to enforce standards effectively, consistent with international best practices [20, 21]. Decentralised regulatory structures can significantly strengthen inspection and law enforcement, suggesting similar potential for Ethiopia.

However, this paper has shown that significant institutional barriers persist. In many regions, tobacco control activities are integrated into broader health department structures rather than housed in independent units, leading to competing priorities and a lack of enforcement focus. This integration also strains human resources, as tobacco control roles are often added to staff members’ existing workloads. This finding is supported by studies conducted elsewhere [22, 23]. Furthermore, the absence of regional implementation guidelines and directives significantly hinders the operationalisation of national smoke-free laws. At the time of this study, only one region had developed this implementation guideline. This may reflect both limited technical capacity at the regional level and a lack of dedicated budgets for policy adaptation. This gap partly explains why the implementation of smoke-free laws was less in many regional towns. These differences also illustrate broader challenges in monitoring decentralised tobacco control laws in Ethiopia, where regions vary widely in terms of technical capacity, resources, and political priorities.

Similarly, a lack of comprehensive and transparent legislation on smoke-free laws and legal frameworks for enforcing smoke-free policies was documented as a barrier in a study conducted in Europe and LMICs [24, 25]. Political leadership and commitment to implementing smoke-free laws extend to some regions, where authorities take decisive action against non-compliant businesses. Examples shared by participants included issuing warnings, imposing fines, and, in some cases, temporarily closing venues operating in violation of the law. Such political commitment is widely recognised as a key determinant of successful tobacco control and supported by studies conducted in Ethiopia and other countries [23, 26, 27]. However, maintaining this level of commitment requires ongoing advocacy to ensure that tobacco control remains a long-term political priority.

Nevertheless, competing national and local priorities such as security concerns, crime, and economic instability often overshadow tobacco control efforts at the regional level. In hard-to-reach areas, particularly those affected by security challenges, the implementation of smoke-free laws has been delayed or deprioritised. By contrast, the relative stability of the capital city created more favourable conditions for more vigorous law enforcement and better performance. The delayed health consequences of tobacco use further reduce its prioritisation compared to more immediate public health threats. These findings support other conclusions in sub-Saharan Africa and elsewhere [28–30]. Socioeconomic factors play a crucial role in the implementation of smoke-free laws. In many regions, cultural and religious norms act as facilitators by discouraging smoking and framing it as undesirable behaviour. Religious teachings were reported to discourage smoking, thereby contributing positively to compliance, consistent with evidence from various global contexts in which religious beliefs and culture have been leveraged for tobacco control [31, 32]. However, tobacco cultivation remains a viable economic livelihood in some regions, reducing the motivation for strict enforcement. In some areas, tobacco farming is viewed as an essential source of household income, making authorities reluctant to enforce strict measures. These financial concerns mirror findings in other LMICs and Europe, where commercial interests and local economies complicate tobacco control enforcement [33, 34]. As of this study, logistical issues compound challenges, as many hospitality venues operate primarily at night when enforcement resources are limited. Budget constraints for enforcement activities also persist as significant barriers, in line with global observations on underfunded tobacco control efforts [22, 35]. Strong networks among government institutions, CSOs, and international organisations facilitate the implementation of smoke-free laws. This study reported collaborations among multiple sectors, including transport, customs, media, tourism, and sports, that support educational activities and enforcement efforts. Notably, some regions have benefited from significant financial support from international stakeholders, resulting in measurable progress toward creating smoke-free environments. Multisectoral collaboration has been identified as a crucial factor in the success of tobacco control initiatives worldwide [36–38]. However, some of these implementations rely heavily on donor-driven initiatives, raising questions about their long-term sustainability.

Despite these positive networks, gaps remain in intersectoral coordination. Law enforcement agencies outside the health sector often do not view tobacco control as part of their mandate, undermining comprehensive enforcement efforts. This is partly due to the absence of clear mandates or standard operating procedures that define the roles of non-health sectors in enforcement. Similar findings have been observed in other LMICs, emphasising the need for integrated, cross-sectoral approaches to enforcement [38–40]. Efforts to raise public awareness and build knowledge about smoke-free laws have been essential facilitators, particularly in urban centres. Capacity-building initiatives targeting hospitality venue owners and managers have improved compliance, though coverage remains uneven across regions. Public education campaigns are widely recognised as crucial to changing social norms and enhancing compliance with smoke-free laws. This is also documented elsewhere [41].

Nevertheless, significant barriers persist. Many people, including some law enforcement personnel, lack an understanding of the smoke-free laws. Misconceptions about products like shisha, which many do not perceive as tobacco, complicate enforcement. Studies globally have noted similar challenges, with alternative tobacco products often perceived as harmless or socially acceptable, undermining tobacco control efforts [42–44]. Furthermore, a study conducted in Poland showed that the prevalence of smoking was consistently higher among men compared to women throughout the years 2012–2021, mirroring the role of social norms and ineffective public awareness interventions [45].

### Strengths and limitations

This study covered diverse settings across Ethiopia, thereby maximising contextual variability and enriching the findings. The inclusion of perspectives from the federal, regional, and district levels further strengthened the analysis’s comprehensiveness. However, this same variability introduced some inconsistencies. For example, social norms were identified as both facilitators and barriers, depending on the region. Additionally, the study relied solely on key informant interviews, in which most participants serve as focal persons for tobacco control; they may be either under- or over-reporting compliance. Social desirability bias may also have influenced responses, particularly when discussing sensitive topics such as enforcement gaps. Moreover, including hospitality venue owners could have provided deeper insights and enriched the data, particularly regarding practical challenges in implementing smoke-free laws on the ground.

## Conclusions

In conclusion, compliance with smoke-free laws in Ethiopia is supported by a strong legal framework, established organizational structures, committed leadership, prioritization of tobacco control, multisectoral collaboration, and increasing public awareness. However, these facilitating factors are primarily concentrated in the capital, while many regional cities lack context-specific directives and dedicated tobacco control units. In addition, security challenges, competing priorities, and social norms that tolerate smoking in some areas continue to undermine compliance. Advancing tobacco control will require the development of region-specific implementation guidelines, the establishment of dedicated regional workforces and accountable institutional structures, and strengthened multisectoral coordination with clearly defined roles, including greater engagement of non-health enforcement bodies such as police and trade offices. Leveraging Ethiopia’s existing strengths, including strong legislation, committed leadership, active networks, and cultural norms that discourage smoking, will be critical to achieving equitable and comprehensive tobacco control nationwide.

## Supplementary Information

Supplementary Material 1.

## Data Availability

The complete datasets used and analysed in the current study are available from the corresponding author upon reasonable request.
