# Supplementary material for: Compliance with smoke-free laws in hospitality venues in Ethiopia: qualitative insights into barriers and facilitators using the tobacco control theoretical framework
Source: BMC Public Health. 2025 Dec 20;26:2089. doi: 10.1186/s12889-025-26012-w (PMC13348261; doi:10.1186/s12889-025-26012-w)
Supplement: Supplementary file 1 — Supplementary Material 1. [file 12889_2025_26012_MOESM1_ESM.pdf]

Annex II: Key Informant Interview Guide

PARTICIPANT INFORMATION FORM

Please take the time to read this participant information sheet.

I [name] am working for the School of Public Health, Addis Ababa University. The School of Public Health is currently working in partnership with the Development Gateway and is conducting a primary data collection for the Tobacco Control Data Initiative (TCDI) project in Ethiopia. The TCDI project aims to design and develop a national online website with data related to tobacco control that meets the needs of stakeholders within government, academia, and civil society, in partnership with national and regional institutions. One of the aims of this project is to identify the barriers and facilitators for the compliance of monitoring and enforcement of tobacco advertising Promotion and sponsorship (TAPS) and smoke-free environment (SFE) laws in Ethiopia. For this study, we would like to conduct interviews with managers, government, or NGO employees who are involved in the tobacco control policymaking and/or program implementation at the national and/or sub-national levels. The senior project lead is Prof. Wakgari Deressa from Addis Ababa University.

Procedures: If you join us for the interview, we will ask you some questions about the compliance monitoring and enforcement of tobacco advertising promotion and sponsorship (TAPS) and smoke-free environment (SFE) laws in Ethiopia. We want to learn about your experience with the implementation, enforcement and monitoring of the tobacco control laws for bans on TAPS and smoke-free environments. The interview will take about one hour and will be audio-recorded.

Confidentiality and Anonymity

To help me remember our discussion, the interview will be [digitally] recorded, and upon my return to the office, the audio file will be transcribed verbatim. Personal details will be anonymized, and your identity will not be intentionally revealed to anyone. All relevant information, such as audio recordings, personal details and interview transcripts, will be securely stored on a password-protected computer. The contents of the interviews will be analyzed and may also be used in publications, such as journal articles and policy briefings. This may include references and quotations from this interview, which will be anonymised to remove any personal details.

I am asking you and others to participate in this study voluntarily.

Right to Refuse or Withdraw
Your participation in this study is voluntary, and you have the right to refuse participation or decline to answer any questions without any consequences. However, since your views will help the government and partners to improve the performance of tobacco regulatory institutions, I encourage your participation. If, having read this participant information, you agree to be interviewed and for the content of this interview to be used for the purposes outlined above, please sign the consent form.

Cost: You will not pay or receive money for participation.

Rights: If you have any further questions about this evaluation, you can contact Prof. Wakgari Deressa at AAU (Mobile Phone +251-911-483714). If you have questions about your rights as a participant, don't hesitate to get in touch with the Ethiopian Public Health Association Institutional Review Board office, Addis Ababa, Ethiopia. Office Phone: +251-1-416 60 83 / 41 / 88; Mobile Phone: E-mail- epha@telecom.net.et.

If you agree to participate, please sign below to indicate that you have understood what the assessment is about and what your role is. You will be given a copy of the signed consent form

Consent form

1. I confirm that I have agreed to be interviewed for this research, and that extracts may be used as described in the participant information sheet.

2. I confirm that I have agreed to the interview being digitally recorded and transcribed

Signature: ________________________ Date __________________

Data collector name ___________________Signature _______________Date __________

Interview guide

Introduction

- Brief welcome, introduction, overview of the study/purpose of the interview (covering the key points in the information sheet).
- Remind participants that taking part is entirely voluntary, there are no right or wrong answers and that they can choose not to answer specific questions and stop the interview at any time.
- Read out each of the statements from the consent form, ensuring that all statements are understood by the study participants. Give the consent form for the study participants for their signature.

**Date**	|___||___| / |___||___| / |__||___|(dd/mm/yy)
**Facilitator Name**	
**Start time**	|___||___|: |___||___|(hour/min)
**End time**	|___||___|: |___||___|(hour/min)
**Name of electronic file**	

Study participants profile

**Variables**	**Response**
Region	
Name of the city	
Age	
Sex	
Institution/affiliation	
Type of the institution	
Interviewee ID	
Position	
Year of experience	
Role and responsibilities in area of tobacco control law	
Year of experience in tobacco control area	

Section I- Tobacco Control law and implementation in Ethiopia

1- Can you tell me about tobacco control laws in Ethiopia?
  - **Follow up**- What specific things are included in this law?
  - **Follow up**- Who are the main initiators of tobacco control laws in Ethiopia?
  - **Follow up**-What are the most important things considered in the formulation of the laws?
  - **Follow up**- Who were the main actors involved in the formulation and to what extent were local stakeholders involved in the process

2- How do you see the implementation of tobacco control law in Ethiopia?
  - **Follow up-** What are the facilitating factors for the implementation of tobacco control law?
  - **Follow up-** What are the barriers for the implementation of tobacco control law?

Section II-Smoke free laws and implementation at hospitality venues in Ethiopia

1- Barriers and facilitators for compliance of monitoring and enforcement of the smoke-free laws within HV in Ethiopia

Please tell me about smoke free laws within hospitality venues in Ethiopia
  - **Follow up –** What specific things are included in this law?
  - **Follow up**- Who is responsible for implementing this law?

2- Compliance about the smoke free environment (SFE) laws at the hospitality venues (HVs) in Ethiopia.

Please tell me about the compliance of the smoke free laws at the HVs in Ethiopia.

- **Follow up**- Do you have any concerns about the implementation of the laws in Ethiopia?
- **Follow up** - Do you think there is a facilitator for the implementation of SF laws at the HVs from all stakeholders?
  - o *Probe- Cultural and social norms*
  - o *Probe- Existing laws*
  - o *Probe- Monitoring and enforcement*
  - o *Probe- Strong coordinating mechanism*
  - o *Probe- Political leadership and commitment*
  - o *Probe - Stakeholders partnership*
  - o *Probe- How do you think these facilitating factors can be maintained?*
  - o *Probe- Do you think these facilitating factors are effectively utilized? If yes, how? If not, why?*

- **Follow up**- What are the barriers for the compliance of the SFE laws at the HVs in Ethiopia*?*
  - o *Probe*- Lack of political leadership and commitment
  - o *Probe*- Lack of monitoring and enforcement
  - o *Probe* - Lack of coordinating mechanism
  - o *Probe*- Stakeholders relations ship

- *Probe*-Lack of resources
- *Probe*- Lack of awareness
- *Probe- How do you think these barriers can be avoided*?

3- Monitoring and enforcement for compliance of SF laws at the HVs in Ethiopia.

Please tell me about the monitoring and enforcement of SF laws at the HVs in Ethiopia?

- **Follow up**- How does the monitoring and enforcement activity for SF laws at the HVs looks like?
  - *Probe – Who are the involved stakeholders for this activity?*
- **Follow up**- What are the facilitating factors for compliance monitoring and enforcement of SF laws at the HVs?
  - *Probe – Political leadership and commitment*
  - *Probe – A good coordination mechanism*
  - *Probe- Stakeholders commitment*
  - *Probe- Awareness about the law and implementation*
  - *Probe – Clear role for the law enforcers*
  - *Probe- Availability of resources*
  - *Probe- Do you think these facilitating factors can be sustainable? How?*
- **Follow up**- What are the barriers for compliance of monitoring and enforcement of SF laws at the HVs?
  - *Probe- Tobacco industry interference*
  - *Probe- Nature of the work primarily related to nighttime Sale of tobacco products at HVs*
  - *Probe – Law enforcers' high workload*
  - *Probe – Law enforcers/police collaboration with HVs owners*
  - *Probe – Lack of resources*
  - *Probe- Lack of leadership and commitment*
  - *Probe- Unclear role for the law enforcers*
  - *Probe- Do you think these can be avoided? How? Or why not?*

Summarise the key areas covered and ask if this is correct. If there is anything further they would like to add about compliance with monitoring and enforcement of smoke-free environment laws at the hospitality venues in Ethiopia.

Thank them for their time and input!
